# Polarized training has greater impact on key endurance variables than threshold, high intensity, or high volume training

**DOI:** 10.3389/fphys.2014.00033

**Published:** 2014-02-04

**Authors:** Thomas Stöggl, Billy Sperlich

**Affiliations:** ^1^Department of Sport Science and Kinesiology, University of SalzburgSalzburg, Austria; ^2^Department of Health Sciences, Swedish Winter Sports Research Centre, Mid Sweden UniversityÖstersund, Sweden; ^3^Institute of Sport Science, University of WürzburgWürzburg, Germany

**Keywords:** lactate threshold, peak power, peak oxygen uptake, time to exhaustion, work economy

## Abstract

Endurance athletes integrate four conditioning concepts in their training programs: high-volume training (HVT), “threshold-training” (THR), high-intensity interval training (HIIT) and a combination of these aforementioned concepts known as polarized training (POL). The purpose of this study was to explore which of these four training concepts provides the greatest response on key components of endurance performance in well-trained endurance athletes.

**Methods:** Forty eight runners, cyclists, triathletes, and cross-country skiers (peak oxygen uptake: (VO_2peak_): 62.6 ± 7.1 mL·min^−1^·kg^−1^) were randomly assigned to one of four groups performing over 9 weeks. An incremental test, work economy and a VO_2peak_ tests were performed. Training intensity was heart rate controlled.

**Results:** POL demonstrated the greatest increase in VO_2peak_ (+6.8 ml·min·kg^−1^ or 11.7%, *P* < 0.001), time to exhaustion during the ramp protocol (+17.4%, *P* < 0.001) and peak velocity/power (+5.1%, *P* < 0.01). Velocity/power at 4 mmol·L^−1^ increased after POL (+8.1%, *P* < 0.01) and HIIT (+5.6%, *P* < 0.05). No differences in pre- to post-changes of work economy were found between the groups. Body mass was reduced by 3.7% (*P* < 0.001) following HIIT, with no changes in the other groups. With the exception of slight improvements in work economy in THR, both HVT and THR had no further effects on measured variables of endurance performance (*P* > 0.05).

**Conclusion:** POL resulted in the greatest improvements in most key variables of endurance performance in well-trained endurance athletes. THR or HVT did not lead to further improvements in performance related variables.

## Introduction

Athletes participating in endurance sports such as running, cycling, and cross-country skiing integrate four conditioning concepts into their training program to maximize athletic performance. The first conditioning concept is prolonged high-volume low-intensity exercise (HVT). The second is training at or near the lactate threshold (THR); third is low-volume high-intensity interval training (HIIT) and the fourth concept is a combination of the aforementioned concepts known as “polarized” training (POL). There is a debate as to which of these training concepts may be superior in maximizing adaptations and performance.

HVT executed with low (LOW) intensity [approximately 65–75% of peak oxygen uptake (VO_2peak_) <80% of peak heart rate (HR_peak_) or <2 mmol·L^−1^ blood lactate (Laursen and Jenkins, 2002; Seiler and Kjerland, 2006)] and prolonged duration is thought to be a fundamental training concept in preparing for endurance events. This type of exercise improves VO_2peak_ by increasing stroke and plasma volume and induces molecular adaptations for capillary and mitochondrial biogenesis, thereby improving the efficiency of metabolic key components for energy fueling (Romijn et al., 1993; Midgley et al., 2006).

HIIT has revealed great improvements in athletic performance and related key variables of endurance (e.g., time to exhaustion, time trial performance, VO_2peak_, maximal and submaximal running speed, running economy) in both trained and untrained individuals (Laursen and Jenkins, 2002). These improvements were largely due to increases in O_2_ availability, extraction and utilization and the increases in VO_2peak_ (Daussin et al., 2007; Helgerud et al., 2007). A condensed 2 week block of 10–13 sessions of HIIT led to a 7% increase in VO_2peak_ (Stølen et al., 2005).

Training at or close to the lactate threshold (LT) (Faude et al., 2009), referred to as “threshold training,” improves endurance performance, particularly in untrained participants (Denis et al., 1984; Londeree, 1997). However, Norwegian world-class sprint cross-country skiers demonstrated greater training volume close to the LT when compared to national-level skiers (Sandbakk et al., 2011). Furthermore, in elite cross-country skiers greater improvements in running speed at lactate threshold and performance in a 20-min run when exercising at an intensity eliciting 3–4 mmol·L^−1^ lactate compared with low intensity training (<3–4 mmol·L^−1^) were found (Evertsen et al., 2001). In contrast, experimental and correlational data from well-trained athletes suggest that training time close to LT may be ineffective, or even counterproductive (Esteve-Lanao et al., 2007; Guellich and Seiler, 2010).

Retrospective analysis of the intensity, duration and frequency of the training load of international-level cross-country skiers (Seiler and Kjerland, 2006), rowers (Steinacker et al., 1998), cyclists (Schumacher and Mueller, 2002), and runners (Billat et al., 2001; Esteve-Lanao et al., 2005) revealed that elite endurance athletes completed most of their yearly training sessions at either intensities below (~75% of total training volume) or well above (~15–20% of total training volume) their LT. Six weeks of cycling using POL resulted in greater systemic adaptation in already well-trained athletes when compared to THR (Neal et al., 2013). However, no study has investigated the POL concept in well-trained endurance athletes to determine whether this concept may be superior to the aforementioned training strategies.

In many endurance sports, five key variables have been used as a benchmark to compare athletic performance in and between endurance athletes: (i) VO_2peak_ (Bassett and Howley, 2000); (ii) velocity/power output at the lactate threshold (V/P_LT_) (Bassett and Howley, 2000; Midgley et al., 2007; Faude et al., 2009); (iii) work economy (Di Prampero et al., 1986; Helgerud et al., 2001); (iv) peak running velocity or power output (V/P_peak_) (Midgley et al., 2007); and (v) time to exhaustion (TTE) (Laursen and Jenkins, 2002). The aim of this study was to compare the effects of four training concepts (HVT vs. THR vs. HIIT vs. POL) on the aforementioned key variables of endurance performance in well-trained athletes. We hypothesized that the POL and HIIT group would lead to superior improvements compared with HVT and THR.

## Materials and methods

### Participants

Forty eight healthy competitive endurance athletes who participated in either cross-country skiing, cycling, triathlon, middle—or long-distance running volunteered to take part in this study (mean ± SD: age: 31 ± 6 years, body mass: 73.8 ± 9 kg, height: 180 ± 8 cm). All participants were well-trained [62.6 ± 7.1 mL·min^−1^·kg^−1^ (range: 52–75 mL·min^−1^·kg^−1^)] athletes, accustomed to a workload of more than five sessions per week (10–20 h·wk^−1^) and had frequently been involved in endurance competitions for at least 8–20 years. Participants were members of the Austrian cross-country skiing national team (*n* = 8), running (*n* = 21), triathlon (*n* = 4) or cycling (*n* = 15) teams during or since the year before the current study. Retrospective analysis of training protocols over 6 months prior to the study revealed that none of the participants had regularly executed HIIT. All had followed a HVT training protocol with a maximum of two THR training sessions per week.

Based on the participants' baseline VO_2peak_ and training mode (running or cycling), all athletes were randomized into HIIT, HVT, THR or POL. At baseline, the four groups were not statistically different with regard to age, height, body mass or VO_2peak_. During an initial visit, study details and participation requirements were explained, and written informed consent was obtained. The study received approval from the University of Salzburg Austria Ethics Committee and was conducted in accordance with the Declaration of Helsinki.

### Design

The intervention lasted 9 weeks plus 2 days of pre- and post-testing. All athletes (*n* = 15 cyclists; *n* = 3 triathletes) engaging in cycling training trained with their own bike and completed all tests on a bicycle ergometer (Ergoline, Ergoselect 100P; Bitz, Germany) using their own cycling shoes and pedal system. Other athletes (*n* = 16 runners, *n* = 6 triathletes, *n* = 8 cross-country skiers) ran during the study and completed their pre- and post-testing on a motorized treadmill (HP Cosmos, Saturn, Traunstein, Germany). All participants were instructed not to change their diet throughout the training period and to maintain strength training, if it was part of their training program. Participants' nutritional intake was not standardized or controlled during the study, but for the 3 h prior to all testing in which food intake was not permitted. The training intensity was controlled by HR based on the baseline incremental test: (i) LOW (HR at blood lactate value <2 mmol·L^−1^); (ii) LT (HR corresponding to a blood lactate of 3–5 mmol·L^−1^); (iii) HIGH (>90% HR_peak_)]. The HR was measured during each training session and athletes documented training mode, exercise duration, and intensity in a diary. As a control and for detailed analysis, HR for all training sessions was stored digitally and analyzed retrospectively.

### HVT intervention

**Figure 1:**
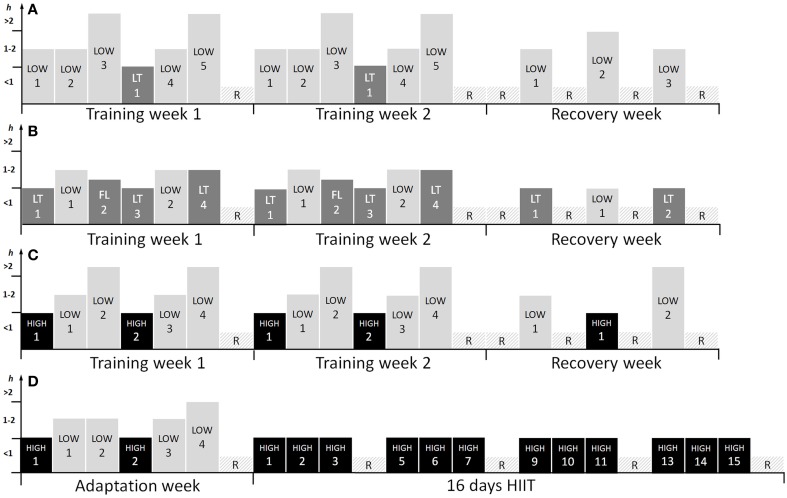
**Training program for 3-weeks of (A) high volume (HVT), (B) threshold (THR), (C) polarized (POL) training, and (D) the training program for the first block of high intensity interval training (HIIT), excluding the recovery week.** LOW, low training intensity (<2 mmol·L^−1^); LT, training intensity around the lactate threshold (3–5 mmol·L^−1^); FL, fartlek; HIIT, high intensity interval training (>90% HR_peak_); R, recovery day.

The HVT included three blocks each lasting 3 weeks: 2 weeks of high-volume training followed by 1 week of recovery (Figure 1A). The two high volume weeks each included six training sessions with three 90 min LOW sessions, two 150–240 min LOW sessions (according to the training mode: running, cycling, or roller skiing) and one 60 min LT session using different types of interval training (e.g., 5 × 7 min with 2 min recovery, 3 × 15 min with 3 min recovery). The recovery week included three training sessions with two 90 min LOW sessions and one 150–180 min LOW session.

### THR intervention

The THR included three blocks, each lasting 3 weeks: 2 weeks of high volume and intensity training followed by 1 week of recovery (Figure 1B). The two high volume and intensity weeks each included six training sessions with two 60 min interval sessions at the LT (5 × 6 min and 2 min recovery in the first block, 6 × 7 min in the second block and 6 × 8 min in the last block), one 90 min LT session with longer intervals (3 × 15 min with 3 min active recovery in the first block and 3 × 20 min for the remaining two blocks), one 75 min session with varying changes in intensity (“fartlek”) (intensities resulting in a blood lactate of 1.5–5 mmol·L^−1^) and two 90 min LOW sessions. The recovery week included one 60 min LOW session and two 60 min LT interval sessions (5 × 6 min with 2 min of active recovery).

### HIIT intervention

The HIIT included two interval blocks of 16 days with one adaptation week prior to and one recovery week after each block. The adaptation week included two 60 min HIIT sessions, three 90 min LOW sessions, one 120 min LOW session and 1 day of recovery. The condensed 16 day interval block included 12 HIIT sessions within 15 days, integrating four blocks of three HIIT sessions for three consecutive days followed by 1 day of recovery. The recovery week contained four LOW sessions of 90 min and 3 days without any training (not presented in Figure 1D). All of the HIIT sessions included a 20 min warm-up at 75% of HR_peak_, 4 × 4 min at 90–95% of HR_peak_ with 3 min active recovery and a 15 min cool-down at 75% HR_peak_ based on the protocol proposed earlier (Helgerud et al., 2007). The LOW sessions lasted 90–150 min depending on the training mode (running vs. cycling) at an intensity resulting blood lactate of <2 mmol·L^−1^.

### POL intervention

The POL included three blocks, each lasting 3 weeks: 2 weeks of high volume and intensity training followed by 1 week of recovery (Figure 1C). The high volume and intensity week included six sessions with two 60 min HIIT sessions, two 150–240 min long duration LOW sessions (duration according to training mode: cycling, running or roller skiing), which included six to eight maximal sprints of 5 s separated by at least 20 min, and two 90 min LOW sessions. The recovery week included one 60 min HIIT session, one 120–180 min LOW session and one 90 min LOW session.

### Pre and post-testing

All participants were asked to report well-hydrated and to refrain from consuming alcohol and caffeine for at least 24-h, as well as from engaging in strenuous exercise at least 48-h prior to testing. The pre- and post-tests included the determination of body mass, an incremental test protocol, a work economy and VO_2peak_ ramp protocol.

On the first day participants performed an incremental test on a treadmill (7.2 km·h^−1^; increment: 1.8 km·h^−1^every 5 min, with 30 s recovery between stages, inclination 1%) or cycle ergometer (80 W; increment: 40 W every 5 min, cadence >80 rpm) until volitional exhaustion was achieved to assess the peak velocity/power output (V/P_peak_), HR, blood lactate, as well as the velocity, power output and HR at 2 and 4 mmol·L^−1^ blood lactate (V/P_2_, V/P_4_ and HR_2_, HR_4_). The participants' HR was recorded by telemetry (Suunto t6, Helsinki, Finland) at 2-s intervals. The mean HR over the last 30 s of each increment was used for statistical analysis. A 20 μl blood sample from the right earlobe was collected immediately after each increment, as well as 3 and 5 min after the completion of the test into a capillary tube (Eppendorf AG, Hamburg, Germany). All samples were analyzed amperometric-enzymatically (Biosen 5140, EKF-diagnostic GmbH, Magdeburg, Germany) in duplicate, and the mean of the two measures was used for statistical analysis. The lactate sensor was calibrated before each test using a lactate standard sample of 12 mmol·L^−1^. Results within a range of ± 0.1 mmol·L^−1^ were accepted.

One day after the incremental tests, all athletes completed a combined work economy and VO_2peak_ ramp protocol to determine their submaximal and peak VO_2_ (VO_2submax_ and VO_2peak_) and HR (HR_submax_ and HR_peak_), as well as time to exhaustion (TTE). First, the intensity for running was set at 8 km·h^−1^ (inclination: 5%) on the treadmill, and for cycling at 200 W with a cadence of >80 rpm for 10 min to determine VO_2submax_ and HR_submax_ for this intensity. The mean VO_2_ and HR during the last 5 min of these tests were used for statistical purposes. The intensity was then increased every 30 s by 0.5 km·h^−1^ (inclination: 10%) on the treadmill or 15 W on the cycle ergometer until exhaustion. The overall time for the ramp test was defined as time to exhaustion (TTE). VO_2_ was measured with an open circuit breath-by-breath spirograph (nSpire, Zan 600 USB, Oberthulba, Germany), which was calibrated prior to each test using high precision gas (15.8% O_2_, 5% O_2_ in N; Praxair, Düsseldorf, Germany) and a 1L syringe (nSpire, Oberthulba, Germany). All respiratory data were averaged every 30 s. VO_2peak_ was achieved if three of the four following criteria were met: (1) plateau in VO_2_, i.e., an increase < 1.0 mL·min^−1^·kg^−1^ despite an increase in velocity or power output; (2) respiratory exchange ratio >1.1; (3) HR ± 5% of age predicted HR_peak_; and (4) peak blood lactate (LA_peak_) > 6 mmol·L^−1^ after exercise. Reliability analysis of The VO_2peak_ test (*n* = 18) revealed ICC values of 0.96 for VO_2peak_ and 0.98 for TTE.

### Statistical analyses

All data exhibited a Gaussian distribution verified by the Shapiro-Wilk's test and, accordingly, the values are presented as means ± *SD*. Two-Way 2 × 4 repeated-measures ANOVA (2 times: pre–post, 4 groups) to test for global differences between pre- and post-intervention, the four training groups and the interaction effect between both factors was applied. When a significant main effect over time was observed, paired *t*-tests within each group were conducted. Based on the different units of peak power/velocity and power/velocity at 2 and 4 mmol·L^−1^ blood lactate in the incremental and VO_2peak_ test, percent changes between pre- to post-values were calculated, and a One-Way ANOVA between groups was performed using Tukey's *post-hoc* analysis. Furthermore, within group changes for these variables were calculated using Wilcoxon tests. An alpha value of <0.05 was considered significant. The Statistical Package for the Social Sciences (Version 20.0; SPSS Inc., Chicago, IL, USA) and Office Excel 2010 (Microsoft Corporation, Redmond, WA, USA) were used for statistical analysis.

## Results

Forty-one participants completed the 9 week training protocol, fulfilling more than 95% of the training program and staying within the given HR zones. Seven subjects (2 in HIIT, 1 in HVT and 4 in THR) withdrew from the study due to illness (*n* = 2), or were excluded due to changes in competition schedule (*n* = 3) or for not fulfilling the training protocol (*n* = 2). The total training hours, number of training sessions and their percent distribution within LOW, LTP, and HIIT are presented in Table 1. POL and HVT had higher training volume compared with THR and HIIT (*P* < 0.05–0.001), while having a similar number of training sessions (*P* > 0.05). HVT demonstrated the greatest amount of LOW, THR of LT, and HIIT in HIGH training sessions (all, *P* < 0.05).

**Table 1:** **The distribution of volume and training intensity within the 9 weeks training intervention (excluding strength training)**.

	**POL**	**HIIT**	**THR**	**HVT**	***F*-Value**	***P*-Value**
Total hours	104 ± 20^‡^^$^	66 ± 1^*^	84 ± 7^*^	102 ± 11^‡^^$^	^a^*F*_(3, 37)_ = 20	<0.001
Number of sessions	54 ± 3	47 ± 1	49 ± 3	58 ± 3	^a^*F*_(3, 37)_ = 1.6	n.s.
Amount of training at low intensity (%)	37 ± 9 (68 ± 12%)^*^	20 ± 1 (43 ± 1%)^†^^§^	23 ± 6 (46 ± 7%)^†^^§^	49 ± 7 (83 ± 6%)^*^	^a^*F*_(3, 37)_ = 41	<0.001
Amount of training at lactate threshold (%)	3 ± 4 (6 ± 8%)^*^	0 (0%)^*^	26 ± 2 (54 ± 7%)^*^	9 ± 3 (16 ± 6%)^*^	^a^*F*_(3, 37)_ = 197	<0.001
Amount of training at high intensity (%)	14 ± 3 (26 ± 7%)^*^	27 ± 1 (57 ± 1%)^*^	0 (0%)^†^^‡^	1 ± 1 (1 ± 1%)^†^^‡^	^a^*F*_(3, 37)_ = 769	<0.001

The values presented are means ± SD. F- and P-values were obtained by One-Way ANOVA (4 training groups). POL, polarized training group; HIIT, High intensity interval training group; THR, threshold training group; HVT, high volume training group.

* Different from all other groups.

† Different from training group “POL.”

‡ Different from training group “HIIT.”

$ Different from training group “THR.”

§ Different from training group “HVT.”

a Main effect between groups.n.s., not significant.

Body mass after HIIT was reduced by 3.7 ± 3.0% (baseline: 73.5 ± 6.8 kg, post: 70.7 ± 6.5 kg, *P* < 0.01) with no significant change in the HVT, THR or POL groups. The reduction in body mass after HIIT was greater compared to other training interventions (*P* < 0.001).

Percent changes in variables from pre- to post-training and between the training concepts during the VO_2peak_-ramp, work economy, and incremental tests are presented in Table 2. POL demonstrated the greatest increase in VO_2peak_ with an 11.7 ± 8.4%, (60.6 ± 8.3–67.4 ± 7.7 ml·min^−1^·kg^−1^; *P* < 0.001), followed by HIIT with a 4.8 ± 5.6% increase (*P* < 0.05). The change in VO_2peak_ in POL was higher compared to THR and HVT (*P* < 0.001 and *P* < 0.05). Absolute VO_2peak_ increased in POL by 10.4 ± 7.9% (*P* < 0.001), which was greater compared with the other training concepts (HIIT and HVT *P* < 0.05, THR *P* < 0.001). No changes from pre to post and no differences between training groups with respect to HR_peak_, LA_peak_, and HR_2 & 4_ were detected (*P* > 0.05). Work economy increased following HIIT (−6.7 ± 4.4% decrease in HR, *P* < 0.01) and THR (−2.7 ± 1.0% decrease in HR, *P* < 0.05) with no significant differences between the other concepts. Work economy expressed as percent of VO_2peak_ was only improved after POL (−4.8 ± 7.6%, *P* < 0.05) with no significant differences between the other training groups.

**Table 2:**
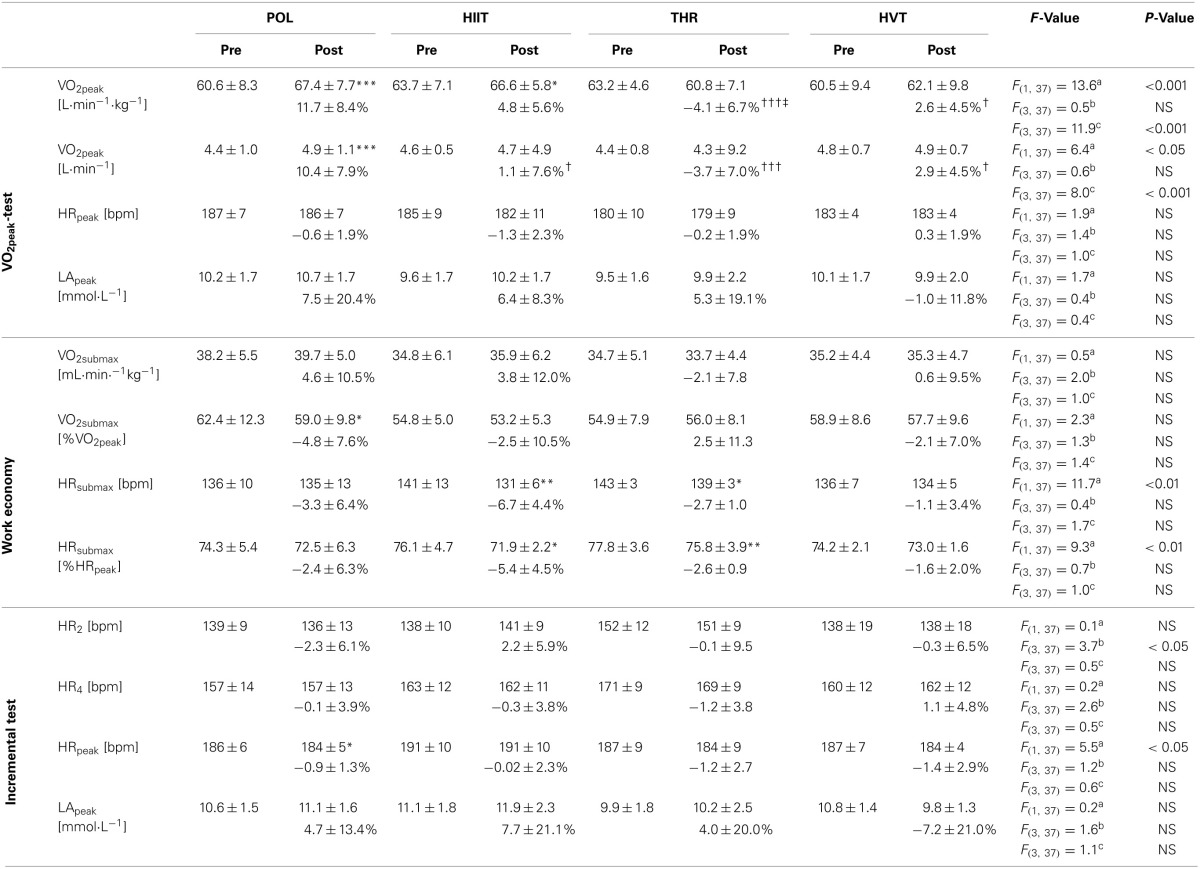
**Changes in physiological variables from pre- to post-training**.

The values presented are means ± SD. F and P values were obtained by Two-Way ANOVA (*2* × *4*: time × training group) with repeated measures. POL, polarized training group; HIIT, High intensity interval training group; THR, threshold training group; HVT, high volume training group; VO_2_, oxygen uptake; HR, heart rate; LA_peak_, peak blood lactate concentration; ^*^p < *0.05*; ^**^p < *0.01*; ^***^p < *0.001* significant difference within groups from pre- to post-training. ^†^*p* < *0.05*; ^†††^*p* < *0.001* significant different from POL training group.^‡^*p* < *0.05* significant different from HIIT training group. ^a^Main effect between pre- and post-test. ^b^Main effect between training groups. ^c^time × training group interactive effect.

The changes in TTE, V/P_peak_ and V/P_2 & 4_ from pre- to post-training and between the single training groups are presented in Table 3. The largest percentage increase in TTE, assessed using the VO_2peak_ ramp test, was observed in response to POL (+17.4%, *P* < 0.001) followed by HIIT (+8.8%, *P* < 0.01); however, no statistical differences were found between the four training concepts. V/P_peak_ in the incremental test increased in response to POL and HIIT (5.1 ± 3.0% and 4.4 ± 2.8%, both *P* < 0.01) with both groups demonstrating greater changes than HVT (*P* < 0.01 and *P* < 0.05). V/P_4_ increased after POL (8.1 ± 4.6%, *P* < 0.01) and HIIT (5.6 ± 4.8%, *P* < 0.01) demonstrating greater changes after POL compared to THR and HVT (both *P* < 0.05).

**Table 3:** **Per cent changes in velocity (V) and power (P) and at various lactate thresholds as well as peak velocity and power**.

	**POL**	**HIIT**	**THR**	**HVT**	***F*-Value**	***P*-Value**
TTE	17.4 ± 16.1^***^	8.8 ± 8.6^**^	6.2 ± 9.0	8.0 ± 10.3	^a^*F*_(3, 37)_ = 2.0	NS
V/P_2_	9.3 ± 12.4	12.1 ± 8.8^**^	2.0 ± 13.8	0.8 ± 13.3	^a^*F*_(3, 37)_ = 1.9	NS
V/P_4_	8.1 ± 4.6^**^	5.6 ± 4.8^*^	1.4 ± 4.3^†^	1.2 ± 6.6^†^	^a^*F*_(3, 37)_ = 4.5	<0.01
V/P_peak_	5.1 ± 3.0^**^	4.4 ± 2.8^**^	1.8 ± 4.8	−1.5 ± 4.9^††^^‡^	^a^*F*_(3, 37)_ = 4.6	<0.01

The values presented are means ± SD. F and P values were obtained by One-Way ANOVA (4 training groups) calculated over the per cent differences between pre- to post-training. POL, polarized training group; HIIT, High intensity interval training group; THR, threshold training group; HVT, high volume training group; TTE, time to exhaustion during the ramp test; V/P_*2*_, velocity or power at 2 mmol·L^-*1*^; V/P_*4*_, velocity or power at 4 mmol·L^-*1*^; V/P_peak_, peak velocity or power in the incremental test;

* *p* < *0.05*;

** *p* < *0.01*;

*** *p* < *0.001 significant difference within groups from pre- to post-training.*

† *p* < *0.05*;

†† *p* < *0.01 significant different from POL training group.*

‡ *p* < *0.05significant different from HIIT training group.*

a Main effect between groups.

## Discussion

The purpose was to determine whether HIIT, HVT, THR, or POL provides the greatest impact on key variables of endurance performance in well-trained athletes. The main findings were that (1) POL led to the greatest improvement in VO_2peak_, TTE and V/P_peak_; (2) V/P_4_ increased after POL and HIIT; (3) no significant differences in work economy were observed pre to post between any of the groups; and finally (4) body mass decreased by 3.7% in response to HIIT.

There are several challenges associated with conducting an exercise training intervention such as the one presented here. Firstly, the compliance of all athletes is paramount to the successful completion of the study and for the subsequent examination of the intervention. The athletes attended more than 95% of all training sessions and all completed their predetermined training load (intensity based on HR zones, duration, and frequency), which was confirmed by logging the daily training dose in a diary and retrospective analysis of HR data. Secondly, an experimental study is difficult to conduct in elite athletes because typically neither the athletes nor their coaches like to have the athletes' training intensity, duration or frequency altered. However, we successfully managed to conduct the current study in well-trained male and female athletes (VO_2peak_: 52–75 mL·min^−1^·kg^−1^) over a 9 week period.

Of the four training concepts, POL resulted in the greatest increase in VO_2peak_, TTE,V/P_peak_ and, together with HIIT, in V/P_4_. As mentioned, POL was confirmed by retro-perspective analysis of the intensity, duration and frequency distribution of the training load in highly trained athletes (Steinacker et al., 1998; Billat et al., 2001; Schumacher and Mueller, 2002; Seiler and Kjerland, 2006; Esteve-Lanao et al., 2007). In these studies, it was demonstrated that endurance athletes perform approximately 75% of their yearly training program either below or well above (~15–20%) the LT, but little at the LT. In the current study, POL mimicked this distribution (LOW = 68%, LTP = 6%, HIGH = 26%). Only the study of Neal et al. (2013) demonstrated that 6 weeks of POL resulted in greater systemic adaptation in trained cyclists when compared to THR, hence supporting our findings.

In moderately trained persons, HVT improves metabolic and hemodynamic adaptations over 3 days (Green et al., 1987, 1990; Coyle, 1999). However, a greater volume of training (~3–5 weeks with 3–5 sessions·wk^−1^) is needed to improve VO_2peak_ (Laursen and Jenkins, 2002). One reason due to why athletes may choose a high amount of HVT may be due to that HVT leads to improved fat and glucose utilization (Romijn et al., 1993), which is beneficial for long lasting endurance events. Therefore, it might be reasonable to implement HVT in the training programs of elite endurance athletes for improving oxidative flux, which is important for converting energy aerobically and recovery after and during HIIT sessions with large anaerobic portions. When HVT becomes the major component of a training program and HIIT sessions are neglected, no further improvement in VO_2peak_ and performance in already well-trained athletes occur (Costill et al., 1988; Laursen and Jenkins, 2002); in line with the findings of the present study. Further improvements of well-trained athletes require adding high intensity training sessions to HVT, as demonstrated in POL. However, due to that the participants of this study mainly used HVT prior to this experiment, the HVT model might not have provided an adequate stimulus for further adaptations.

The advantage of HIIT compared to HVT lies in a shorter period of training time for similar muscular adaptations (Gibala et al., 2006; Burgomaster et al., 2008). In response to HIIT, several central and peripheral adaptations including increased stroke (Helgerud et al., 2007) and blood volume (Shepley et al., 1992), O_2_ extraction (Daussin et al., 2007), and improvements in aerobic and anaerobic metabolism (Macdougall et al., 1998), such as increased mitochondrial biogenesis and oxidative capacity, have been reported (Gibala et al., 2006; Daussin et al., 2007, 2008; Burgomaster et al., 2008). The aforementioned adaptations in response to HIIT explain the often documented increases in TTE, time trial performance (Lindsay et al., 1996), lactate and ventilatory threshold (Acevedo and Goldfarb, 1989; Edge et al., 2005) and VO_2peak_ (Laursen and Jenkins, 2002; Gibala et al., 2006; Midgley et al., 2006; Daussin et al., 2007, 2008; Burgomaster et al., 2008).

The present study, as well as that of Helgerud et al. (2007), demonstrated that training at or near VO_2peak_ may be more effective in enhancing VO_2peak_ when compared to HVT or THR. However, POL, a combination of HVT and HIIT, may be superior for enhancing VO_2peak_ and performance. Numerous studies using “blocked” or “condensed” HIIT (i.e., several HIIT session in 1 or 2 weeks) aim to increase VO_2peak_ (Stølen et al., 2005). Furthermore, in HIIT intervention studies (2–3 HIIT sessions per week), VO_2peak_ increased approximately 9% over a 10 week training intervention (McMillan et al., 2005) and 11% over a 6 week training intervention (Helgerud et al., 2001) in youth and junior soccer players, suggesting a 0.5% increase in VO_2peak_ per HIIT training session. In the current study, the increase in VO_2peak_ following 9 weeks of HIIT (27 HIIT sessions) was 4.8% (0.18% increase per training session), while POL resulted in an 11.7% increase in VO_2peak_ with fewer HIIT sessions (14 HIIT) (0.84% increase per training session). This result may be explained by: (1) peak adaptation might have been reached following the first HIIT block, and therefore, repeated HIIT bouts did not produce any further improvements in VO_2peak_ or performance, or (2) the combination of HVT and HIIT, much like in POL, leads to greater long-term adaptations in endurance performance than with exclusively HIIT or HVT.

THR improves VO_2peak_, lactate or ventilatory thresholds and endurance performance in untrained persons (Denis et al., 1984; Londeree, 1997; Gaskill et al., 2001). These findings contrast those of the current study, as we did not observe improvements in VO_2peak_, V/P_4_, TTE or V/P_peak_ in our elite athletes in response to THR. Additionally, it is possible that in well-trained endurance athletes, repeated training bouts at LT might generate unwarranted sympathetic stress (Chwalbinska-Moneta et al., 1998), while offering no further stimulus for performance enhancement (Londeree, 1997). In this context, especially within the THR group, significant variability in the individual changes in VO_2peak_ from pre- to post-intervention were observed (range: −20 to +4%). However, some THR training might be beneficial to well-trained athletes since world-class sprint cross-country skiers demonstrated greater training volume in the low and moderate intensity zones compared with national-level skiers (Sandbakk et al., 2011).

The body mass of the well-trained athletes decreased by 3.7% (approximately 3 kg) after HIIT, but not in response to the other training concepts. HIIT favors lipid oxidation and promotes adipose tissue loss (Perry et al., 2008; Boutcher, 2011). Depending on the athlete's baseline value, reduction in body mass may negatively impact immune function and overall health, as well as induce a catabolic state. Training blocks with increased volume and/or exercise intensity might induce symptoms of “overreaching,” reduced physical capacity, burnout symptoms including tiredness, and lack of energy (Angeli et al., 2004). However, despite the large differences in the individual responses in some of the training groups, none of the athletes demonstrated reduced TTE or V/P_peak_ after the study, nor did they report any of the aforementioned symptoms during and after the 9 week intervention. Based on the observed changes in body mass and smaller increases in VO_2peak_ in the HIIT group compared with previously published data (McMillan et al., 2005; Stølen et al., 2005; Helgerud et al., 2007), longer blocks of training periods with high intensities could provoke these symptoms.

Except for significant decreases in %VO_2__peak_ in the POL group and HR_submax_/%HR_peak_ in the HIIT and THR groups (with no group differences), no improvements in work economy were found in the current study. Helgerud et al. (2007) reported a ~5% improvement in running economy after THR, HIIT, and HVT with no differences between groups. These improvements were mainly attributed to an increased amount of running training. Therefore, the applied training regimes were largely responsible for the changes in V/P_peak_and VO_2peak_, while work economy remained fairly constant. V/P_4_ was only improved in POL (8.1%) and HIIT (5.6%). This is consistent with findings demonstrating that running velocity at lactate threshold follows the improvements in VO_2peak_ (Helgerud et al., 2001; McMillan et al., 2005).

## Limitations and perspectives

Standardized methodology of performance diagnostics (incremental test and VO_2peak_ ramp protocol) was utilized to evaluate the effects of the four endurance training interventions on key variables of endurance performance. However, a direct transfer to specific competition situation (e.g., time trial) need to be established in future research. Furthermore, the increase of about 11% in VO_2peak_ with POL within 9 weeks is large for well-trained endurance and has to be put in perspective within the annual training periodization. Long-term training studies are warranted to evaluate these aspects.

## Conclusion

In this study of elite athletes performing HIIT, HVT, THR or POL training, POL results in the greatest improvements in key variables of endurance performance (VO_2peak_, TTE, V/P_peak_, and V/P_4_). HIIT led to a decrease in body mass and less pronounced increases in VO_2peak_ compared with previous findings using short term (1–2 weeks) HIIT, suggesting that a 9 week HIIT should be applied with care. Exclusive training with THR or HVT did not lead to further improvements in endurance performance related variables in well-trained athletes.

## Disclosure of funding

No funding was received for this work from the National Institutes of Health, the Welcome Trust, the Howard Hughes Medical Institute, or other funding agencies to PubMed Central. None of the authors had any professional relationships with companies or manufacturers who will benefit from the results of the present study. The authors declare no conflict of interest.

## Author contributions

Conception and design of the experiments: Thomas Stöggl, Billy Sperlich. Performance of the experiments: Thomas Stöggl. Data analysis: Thomas Stöggl, Billy Sperlich. Preparation of the manuscript: Thomas Stöggl and Billy Sperlich. Both authors read and approved the final manuscript.

### Conflict of interest statement

The authors declare that the research was conducted in the absence of any commercial or financial relationships that could be construed as a potential conflict of interest.
